# The role of leadership among a Congolese community in Australia in response to the COVID-19 pandemic: a narrative study

**DOI:** 10.5365/wpsar.2022.13.2.914

**Published:** 2022-06-24

**Authors:** Sunita J Rebecca Healey, Nafiseh Ghafournia, Katarzyna Bolsewicz, Karinne Andrich, Peter D Massey

**Affiliations:** a Hunter New England Health, Wallsend, New South Wales, Australia.

## Abstract

**Objective:**

Community leadership enhances collective action in times of uncertainty, such as during the coronavirus disease (COVID-19) pandemic. This study explores the role of leadership related to the COVID-19 response and information sharing among a newly emerging Congolese community in the Hunter New England region of Australia.

**Methods:**

Semi-structured qualitative inquiry was used to interview four participants who were identified as being influential leaders of the local Congolese community. The findings of this study were part of a larger exploration of COVID-19 messaging among emerging culturally and linguistically diverse (CALD) communities. Two interviewers independently analysed the transcribed data before pairing their findings. Narrative analysis was employed.

**Results:**

Two major themes were identified: leadership as an assigned and trusted role, and leadership as a continuous responsibility. Several categories were identified within these themes, such as mutual connection, education level, multilingual ability and networking.

**Discussion:**

The Congolese community leaders reported feeling responsible and confident in their ability to proactively contribute to the local COVID-19 response by enhancing communication within the community. By partnering with and learning from respected leaders in CALD communities, government health services have the opportunity to improve how current public health messaging is developed.

In Australia, collaboration with leaders of culturally and linguistically diverse (CALD) communities has been recommended for communication of coronavirus disease 2019 (COVID-19) health information, particularly around vaccination. (*1*, *2*) Many refugee communities are tightly knit and rely on advice and guidance from community leaders. (*3*)

Congolese immigrants are a newly emerging population in regions of Australia, such as the Hunter New England area, with most coming to Australia as humanitarian arrivals. Refugees from the Democratic Republic of the Congo have been subject to human rights violations related to the country’s history of intermittent armed conflict and political unrest. (*4*, *5*) Most refugees from the Democratic Republic of the Congo crossing the border reach rural settlements or camps in neighbouring Burundi, Rwanda, United Republic of Tanzania and Uganda, where work and educational opportunities are limited. (*5*) Additionally, basic humanitarian needs have been further compromised by multiple Ebola virus disease outbreaks since 2018. (*4*) Many Congolese are multilingual, but it is estimated that less than 50% of Congolese refugees speak English, and only 10% report the ability to read and write English well. (*5*)

By listening to community leaders, we explored and gained insights into the concept of leadership among a newly emerging Congolese population in Australia, and how leadership is being enacted during the COVID-19 response. No similar work has been reported in Australia or internationally.

## Methods

Four participants of Congolese or Burundian background were recruited by purposive sampling among community leaders, after initial recommendation by a local refugee health nurse. The participants took part in semi-structured interviews as part of a larger project. Three participants were interviewed together and one individually.

Researchers from the Multicultural Health and Refugee Health Service (MHRHS) conducted the interviews in English, exploring participants’ roles in relation to COVID-19 information sharing. “Community” was defined as “recently arrived refugee immigrants who identified as having Democratic Republic of the Congo heritage, living in the Hunter New England region.” Participants often chose to broaden this term to include refugees of Central African background living locally. “Leadership” was ascertained by participants themselves. All four participants self-reported being leaders in their community, and this was corroborated by the other participants.

The Congolese have a strong oral tradition that values storytelling as a way of making sense of the world and conveying knowledge. (*6*) Narrative analysis was employed to interpret the roles and experiences through stories. (*7*) Two researchers analysed the data individually before combining to create a paired analysis for each transcript. Researchers focused on the content of the stories and how the narrator organized information to convey meaning. (*8*) Similar concepts were grouped into categories, forming two overarching themes.

## Results

The participants comprised three women and one man, aged between 20 and 60 years. All could read, write and speak both Swahili and English, and several minority languages. Three participants provided additional information, reporting lived experiences as a refugee, attainment of tertiary education and holding employment positions in Australia that were external to their roles with the African community.

Each participant shared stories using a similar structure, where the main point was given first and then explained. The participants circled back to their main points for emphasis and spoke confidently of community and culture.

The narrative structure and the content of the participants’ narratives revealed two aspects of leadership in this resettled Congolese community – that it is a socially assigned and trusted role, and it carries an ongoing responsibility.

### Leadership as a socially assigned and trusted role

Participants told stories of leadership as a role bestowed by community members and of how that role was respected by work colleagues outside the community (**Table 1**). They reported that the community’s perceptions about education level empowered a leader who could research and provide accurate advice and information. Education boosted participants’ self-confidence, as one described: “I’m not the expert, but my level of education allows me to go seek the right level of education [information] that I can also spread.” Being multilingual was advantageous in navigating and understanding media resources in English and other languages, and it provided participants with additional leverage to inquire, interpret and explain COVID-19 information.

**Table 1:** Evidence supporting theme: Leadership as a socially assigned and trusted role

Leadership as a socially assigned and trusted role	Participants’ quotes
Role bestowed by others	Yeah, the day I went to drop my CV and we talking and they say well we know you are a leader, and this is the refugee from there and we know that you help them, so we know you will help us as well... And we work together.
Importance of education and English language skills	The educational background I have back home and here, it added up to people to trust me, people to ask me things … and also the capacity of advocating on their behalf to gain the trust in the community, and also have something to contribute.
	[Community members] contact me because they believe, as a leader, that I have done more research … so they just want to double check, because [for] some [of them] English language may still be low.
Shared language and culture	So it’s just mutual connection, network, one-to-one talk, that helps channel information, and update one another.
	So most times, people would call and say “we heard about this” and ask “how much more did you hear about this”? And then you have to go through it and explain in a language they understand. Because most of us, we share … the same language and background, so it’s an easy task to explain in their local language or in a language they understand better.
Relationship with trust	I strongly believe that my role as a community connector, and also the trust the community has in me, has impacted me to do everything that I can offer to the community.

Mutual cultural understanding, sharing common languages and connection with the community were described by participants as factors contributing to trust and connectivity. One participant explained: “I share the same cultural background as community members, like people from Burundi, and those that speak the languages I use … having supported different people and built trust from the community, it’s from mutual understanding and the relationship that makes me play a key role.” Being trusted by the community strengthened the participants’ confidence to be leaders. One participant explained: “According to that trust, I feel free to interact with people who ask to know something.”

### Leadership as a continuous responsibility

The responsibility of leadership was seen as continual: one participant described “leading the community in our regular gathering,” and another explained that “we usually talk nearly every single day and pass what we have heard in the news in our local languages.” Participants reported carrying various leadership responsibilities in ways that were caring and respectful; this was displayed through words such as “encourage,” “help,” “tell,” “contribute,” “share,” “benefit” and “connect.”

Participants reported how they carried out their leadership responsibilities and mobilized to help communities (**Table 2**). For example, by interpreting and translating key public health messages and pitching them in ways that were understood by community members. One participant reflected: “Most of us, we share the same language and background, so it’s an easy task to explain in their local language or in a language they understand.”

**Table 2:** Evidence supporting theme: Leadership as a continuous responsibility

Leadership as a continuous responsibility	Participants’ quotes
Responsibility as an ongoing phenomenon	Since I came..., I have good relationship with my Congolese community and also African community around... over 10 years to get to know each other, and also to win trust from the community, because we are here to help each other. Wherever help is needed, when I can respond to, I do that’s how … even when an issue arises, it’s easy to interact … the same way of sharing information about COVID.
Responsibility to act	Then we come out with the idea, ok, let us translate. I did the translation in Swahili, then I post on my WhatsApp, then I say ok … just send me inbox and I will send you to read, the safety … about what the meaning of COVID, what you must do, how you going to wash your hands, that stuff like that, the basic stuff.
	You see, we have that burden of getting people to know what is happening, especially about COVID, because there is also a lot of misinformation. So when we get together, or when we have the opportunity to meet with someone, is trying to fix the information that they got that is not right.
Responsibilty to share accurate information	If we come across something that is related to COVID, before you send it out there, you have to do a little bit of research. So you check the correct source – the government, and if it’s something you heard from social media, if you compare it to what the government has is the same, that’s when you get that and post it on the status or post it on the WhatsApp.
	So we need to ask questions, and so we can have answers to those question and be confident with whatever we are saying.
Responsibility to incorporate network	And also the different work, different jobs I do … all this gives me the position and strength to share what I have to contribute to building the community strength.

Participants described proactively using platforms familiar to community members (e.g. WhatsApp). For example, participants called community members during lockdown periods, translated and voice-recorded COVID-19 information and uploaded audio-links to share on social media. One participant explained that their response to act swiftly and dispel misinformation was in part “because as a community leader, my heart is to see people working within the regulation.”

Participants also took responsibility by passing on information to community members only after validating the information. One participant explained: “I like my source to be accurate; before spreading any information, I need to check the government and health website. That way I know.”

Lastly, participants described how networking opportunities allowed COVID-19 information gained from their workplaces to benefit their community. One participant described the strength of their network in this way: “My tentacles reaches [sic] everywhere. So, in terms of getting useful information, it can come from anywhere.”

## Discussion

There is strong evidence supporting CALD leader engagement in health care, (*1*, *2*) but little is known about how community leadership is recognized and developed. Some insights have emerged from this work where participants have become community leaders through social processes and recognition. Commonality of culture and language were reported to be strong contributors to the community’s perception and trust of the participants as leaders. During the COVID-19 pandemic in particular, being strong English speakers afforded our participants opportunities to network across African and non-African spaces, enhancing the flow of important public health messages. As described by the United Nations High Commissioner for Refugees, higher-level education turns students into leaders. (*9*)

The leadership role entrusted to the participants also brought ongoing responsibility and accountability. Participants reported performing leadership roles by seeking out and conveying accurate COVID-19 information to the community in ways that were understood by the community.

Similarly, in the Ebola response, community engagement by health services that actively partnered with respected leaders and other community mobilizers was critical to success. (*10*) During Ebola outbreaks in Central Africa, members of communities bordering other countries trusted local leaders, not government health workers, as a source of information about Ebola. Similarly, the Congolese community in Australia has strong trust in community leaders. Thus, public health solutions should be tailored to communities by listening to and learning from community leaders. (*11*) Furthermore, the Ebola epidemic showed that a shift from the biomedical model for outbreak response towards a more holistic sociobehavioural model is necessary for policy-makers to achieve collective cooperation. (*12*) Such frameworks have been recommended for the COVID-19 pandemic response worldwide. (*10*, *12*)

The thoughtful engagement of Congolese community leaders has also been described in management of sensitive issues such as sexual and reproductive health. (*13*) Community leaders in the Democratic Republic of the Congo hold strong influence over the community’s perception of sexual health, accessing of services, stigma and cultural taboos. (*13*) By engaging community leaders respectfully with two-way discussions, leaders could become advocates for sensitive health issues within their community. (*13*) Respectful collaboration with community leaders allows public health messages to reach and be received by CALD community members in a timely and meaningful way.

The World Health Organization’s COVID-19 global risk communication and community engagement strategy notes that community-centred participatory approaches provide opportunities for governments to support otherwise unreachable marginalized groups. (*10*, *14*) This is done by identifying and collaborating with community leaders, to co-design and coordinate public health responses that are acceptable to the community. (*10*, *12*)

The Congolese community is diverse: the stories and experiences of the four English-speaking leaders interviewed may not represent other leaders in the Hunter New England region and beyond, particularly those with limited English ability. The sociocultural context of communities may also impact the relationship between communities, leaders and governments, especially in settings of rapid change such as this pandemic; hence, governments need to be flexible and engaged. The lead MHRHS researchers are themselves of a CALD background. Therefore, particular stories and focus may have been drawn from the participants, bringing strength and opportunities for deeper conversation. The small number in this study suited the approach of narrative analysis – exploring the contextual stories and opinions of participants interviewed – but is a limitation.

The COVID-19 pandemic has precipitated responses from community leaders to fill a void in public health communication messaging. The Congolese community in this study had access to people who were socially assigned and given responsibilities as leaders. These individuals were educated and multilingual, and had collaborative abilities and common cultural experiences.

By nurturing two-way communication, government health services can learn and improve upon current methods of COVID-19 messaging to reach CALD communities, to further reduce risks to communities. The public health response in a pandemic should be underpinned by partnerships with leaders to reach common goals. Further studies on leadership and engagement with CALD communities are essential.
